# Hemodynamic and Morphological Differences Between Unruptured Carotid-Posterior Communicating Artery Bifurcation Aneurysms and Infundibular Dilations of the Posterior Communicating Artery

**DOI:** 10.3389/fneur.2020.00741

**Published:** 2020-07-24

**Authors:** Jinlong Yuan, Zhenbao Li, Xiaochun Jiang, Niansheng Lai, Xuanzhi Wang, Xintong Zhao, Degang Wu, Jiaqiang Liu, Dayong Xia, Chenlei Huang, Xinggen Fang

**Affiliations:** ^1^Department of Neurosurgery, The First Affiliated Hospital of Wannan Medical College (Yijishan Hospital), Wuhu, China; ^2^Department of Clinical Laboratory, The First Affiliated Hospital of Wannan Medical College (Yijishan Hospital), Wuhu, China

**Keywords:** computational fluid dynamic, hemodynamics, morphological, communicating artery aneurysms, infundibular dilations

## Abstract

**Objective:** Posterior communicating artery bifurcation aneurysms (PcomA-BAs) and infundibular dilations (PcomA-IDs) are found at the junction between the internal carotid artery (ICA) and the posterior communicating artery (PcomA). Several studies found that PcomA-IDs potentially progress to aneurysms and can even rupture. In our clinical practice, digital subtraction angiography (DSA) helps differentiate PcomA-IDs from unruptured PcomA-BAs. However, when PcomA-IDs are >3 mm in diameter or PcomA are absent on DSA, it is challenging to use DSA to differentiate PcomA-IDs from unruptured PcomA-BAs. Hemodynamic and morphological factors are thought to play important roles in the pathogenesis, progression, and rupture of cerebral aneurysms. We compared hemodynamic and morphological differences in unruptured PcomA-BAs and PcomA-IDs to better manage PcomA-IDs.

**Methods:** We included 83 PcomA-IDs and 115 unruptured PcomA-BAs dignosed and measured using DSA from January 2015 to January 2019. Computational fluid dynamics was performed on these patient-specific models reconstructed using axial slices in DICOM format. Clinical, hemodynamic, and morphological factors were compared between the PcomA-IDs and PcomA-BAs. Significant parameters were analyzed using binary logistic regression analysis to identify independent risk factors. Receiver operating characteristic (ROC) analysis was performed on the independent risk factors to acquire cutoff values.

**Results:** One hemodynamic and four morphyological parameters were significantly different between PcomA-IDs and PcomA-BAs: normalized wall shear stress (NWSS), size, the angle between the ophthalmic segment of the ICA and the PcomA (APcomA), the angle between the ophthalmic and the communicating segment of the ICA (AICA) and the diameter of the PcomA (DPcomA). Binary logistic regression analysis showed that small size and DPcomA as well as APcomA were all independent significant factors characterizing the status of PcomA-IDs and the ROC analysis for independent risk factors indicated the cutoff values of size, APcomA, and DPcomA were 3.45 mm, 66.27°, and 1.24 mm, respectively.

**Conclusions:** Size, DpcomA, and ApcomA could independently characterize the status of PcomA-IDs. These might help us better differentiate them from real aneurysms and guide its management.

## Introduction

Infundibular dilations (IDs) are characterized by the following angiographic features: maximum diameter 3 mm, triangular or funnel shape, and lack of an aneurysmal neck (1). The incidence of IDs, ranging from 7 to 25%, increases with age and aneurysms (especially when multiple aneurysms) occurring in families (2, 3). PcomA-IDs, located at the junction of the ICA and PcomA, are the most common IDs. It remains unclear whether a PcomA-ID reflects a pre-aneurysmal state or just a normal anatomical variant. Despite the fact that PcomA-IDs are usually considered normal vascular variants in which all histological mural layers are still preserved, several case reports found that PcomA-IDs potentially develop into PcomA-BAs (4–9). PcomA-BAs, which demonstrate media and internal elastic lamina degeneration in histology, can lead to catastrophic SAH. Although several case reports also indicated that PcomA-IDs could result in SAH directly (10–12), Griffin et al. showed that PcomA-IDs ruptured directly have already had the characteristics of PcomA-BAs (13). For these reasons, it is desirable to make a distinction between PcomA-IDs and unruptured PcomA-BAs, as doing so will allow better management of PcomA dilations.

In our clinical practice, DSA helps to distinguish PcomA-IDs from unruptured PcomA-BAs. PcomA-IDs characterized by DSA should have the following features: triangular or funnel shape, usually <3 mm in diameter, lack of an neck, and the PcomA should arise from the ID apex. For PcomA-BAs, the maximum diameter should be more than 3 mm and the PcomA should be located at the neck or lateral wall of aneurysm. However, when the PcomA-IDs are >3 mm in diameter or the PcomA are not visible (14), it is challenging to use DSA only in order to differentiate PcomA-IDs from unruptured PcomA-BAs.

Hemodynamic factors play important roles in the pathogenesis, progression, and rupture of intracranial aneurysms (IAs) (15–17). The progression of PcomA-IDs into aneurysms may be induced by complicated hemodynamics. For IDs, only two cases of hemodynamic analysis have been reported. Bake et al. analyzed the hemodynamic factors using CFD in four cases of PcomA-IDs, including the distribution of pressure and wall shear stress (WSS), and advised that patients with IDs should be closely observed (18). Ba et al. presented a patient with carotid ophthalmic aneurysms (OA) with concomitant ophthalmic artery infundibulum (19). They found that hemodynamics of areas were susceptible to further progression and rupture might have high relative residence time (RRT), retarding OA blood flow. Nevertheless, these two hemodynamic studies were case reports. In the present study, we performed hemodynamic and morphological comparisons between 83 PcomA-IDs and 115 unruptured PcomA-BAs so as to distinguish the PcomA-IDs and unruptured PcomA-BAs and better manage the PcomA-IDs. To the best of our knowledge, this is the first study of its kind.

## Materials and Methods

The Research Ethics Committee of Wannan Medical College approved this retrospective study; The study was performed according to the Declaration of Helsinki and all participants provided written informed consent. All patients were selected at the Department of Neurosurgery, the First Affiliated Hospital of Wannan Medical College, Wuhu City, China.

### Patient Selection

We retrospectively reviewed our database including imaging data and medical records. Eighty-three consecutive patients with single PcomA-IDs and 115 patients with single unruptured PcomA-BAs were included from Januarey 2015 to December 2019. All patients with a suspected IA were examined using magnetic resonance angiography (MRA) or computed tomography angiography (CTA) and then underwent DSA after admission.

We divided patients into two groups, PcomA-IDs and unruptured PcomA-BAs, and performed a retrospective analysis. Inclusion criteria were as follows: [1] single PcomA-IDs or unruptured PcomA-BAs diagnosed using DSA; [2] the quality of DSA images was adequate for CFD analysis; and [3] informed consent of patients or families was acquired. Exclusion criteria were as follows: [1] unruptured carotid-posterior communicating artery aneurysms were not bifurcation aneurysms, such as carotid-posterior communicating artery fusiform or dissecting aneurysms, and true posterior communicating aneurysms; [2] participation was refused by patients and families; and [3] the quality of DSA images were too poor for CFD analysis.

### Imaging

A total of 198 3D images were obtained using DSA (Siemens, Germany, Artis zee Floor VC14). Rotational angiograms were performed 1-s after a 5-s contrast injection for a total of 15 mL contrast agent and a 360° rotation, which obtained 266 frames. The corresponding 266 images were reconstructed on the syngo X Workplace workstation into 3D modeling, and then it was exported by stereolithography (STL) format. The pulsatile velocity waveform was obtained using transcranial Doppler from a healthy subject. We then captured the flow spectrum envelope to obtain average blood flow velocity waveform in a whole cardiac cycle using MATLAB 14.0 software (MathWorks, Natick, MA, US).

### Patient-Specific Model

STL exported from the workstation was segmented and smoothed in GEOMAGIC STUDIO 12.0 (Geomagic, Morrisville, North Carolina). Then the surface data were imported into ICEM CFD 14.0 (ANSYS, Canonsburg, Pennsylvania, US) to create volume grids for CFD simulations. The maximum element size was 0.3 mm and all surface data were meshed to create ~1 million finite volume tetrahedral element grids with four layers of prism elements for accurate calculation of WSS. The CFX 14.0 (ANSYS Inc.,USA) was then used to solve the flow governing Navier–Stokes formulations with the assumption of laminar, incompressible, and Newtonian blood flow. The vessel wall was set as a rigid wall with no-slip boundary condition. The density and dynamic viscosity of blood were assumed to 1,050 kg/m^3^ and 0.00345 Pas, respectively. A pulsatile velocity profile, derived from transcranial Doppler from a healthy individual, was applied for the inflow boundary condition. The flow waveforms were scaled to achieve a mean inlet WSS of 15 dyne/cm under pulsatile. The outlet was set as an opening boundary condition with zero static pressure. We discretized the entire cardiac cycle of 0.8 s by a time-step of 0.001 s for numeric simulation. Three cardiac cycles were simulated to acquire stable data, and the last cycle was taken as output.

### Hemodynamic Parameter Calculation

Three hemodynamic parameters were calculated: NWSS, OSI (oscillatory shear index), and RRT. NWSS is defined as WSS of the aneurysm wall divided by WSS of the parent artery wall, to allow comparisons among different patients (16, 20). OSI, measuring the directional change of WSS during the cardiac cycle, is a non-dimensional parameter. RRT, which is a combination of WSS and OSI, reflects the residence time of blood flow near the wall.

(1) $$\begin{array}{r} WSS=\frac{1}{T}\overset{T}{\underset{0}{\int }}|wss_{i}|dt \end{array}$$

(2) $$\begin{array}{r} OSI=\frac{1}{2}\left\{1-\frac{\left|\overset{T}{\underset{0}{\int }}wss_{i}dt\right|}{\overset{T}{\underset{0}{\int }}|wss_{i}|dt}\right\} \end{array}$$

(3) $$\begin{array}{rcl} RRT & = & \frac{1}{\left(1-2\times OSI\right)\times WSS}=\frac{1}{\frac{1}{T}|\overset{0}{\underset{T}{\int }}wss_{i}dt|} \end{array}$$

where wss_i_ is the instantaneous WSS vector and T is the duration of the cycle. The OSI was averaged over the dome area.

### Morphological Parameter Calculation

Ten morphological parameters described by Dhar et al. and Yu et al. were included in this study (21, 22). For example, in accordance with Dhar et al., size was defined as the maximum perpendicular height of the Intracranial aneurysm. The maximum perpendicular distance of the dome from the neck plane was measured (21). In addition to 3D morphological parameters such as undulation index (UI), ellipticity index (EI), and non-sphericity index (NSI), other 2D variables were measured using DSA. The 3D morphological parameters calculated by the method of Lv et al. (23). To acquire the accurate result of morphological parameters, two neurosurgeons performed the calculations.

### Statistical Analysis

All data were analyzed using SPSS Statistics version 20.0 (SPSS, Inc., Chicago, Illinois, US). For categorical variables, the χ^2^ test was used to compare the differences. For the normally distributed parameters, data were described as means ± SDs. For abnormally distributed parameters, data were described as deviation medians and IQR. The differences of all parameters between the PcomA-IDs and the PcomA-BAs groups were analyzed using two-tailed independent Student *t*-tests. When *p* < 0.05, the differences were considered statistically significant. Statistically significant variables in univariate analysis were further evaluated using binary logistic regression analysis to identify independent risk factors and ROC analysis was performed on the independent risk factors to acquire cut off values (Figure 2).

## Results

### Clinical Characteristics

Eighty-three patients with single PcomA-IDs and 115 patients with single PcomA-BAs were included. The total of 198 patients included 141 females and 57 males with mean age 57.78 ± 10.63 years in the PcomA-IDs group (29 males and 54 females) and 59.29 ± 8.62 years in the PcomA-BAs group (28 males and 87 females). The clinical characteristics of patients with PcomA-IDs and PcomA-BAs are shown in Table 1. There were no significant differences between groups in terms of age, gender, hypertension, diabetes, drinking, smoking, hyperlipidemia, atherosclerosis, cerebral infarction, or coronary heart disease.

**Table 1:** Clinical characteristics of PcomA-IDs and PcomA-BAs.

**Variable**	**PcomA-IDs (*n* = 83)**	**PcomA-BAs (*n* = 115)**	***P*-value**
Age	57.78 ± 10.63	59.29 ± 8.62	0.290
**Gender**			
Female	54	87	
Male	29	28	0.104
**Hypertension**			
Yes	43	67	
No	40	48	0.367
**Diabetes**			
Yes	**7**	11	
No	76	104	0.785
**Drinking**			
Yes	13	20	
No	70	95	0.747
**Smoking**			
Yes	9	21	
No	74	94	0.151
**Hyperlipidemia**			
Yes	12	25	
No	71	90	0.195
**Atherosclerosis**			
Yes	10	18	
No	73	97	0.473
**CI**			
Yes	6	12	0.439
No	77	103	
**CHD**			
Yes	7	14	0.399
No	76	101	

*CI, Cerebral infarction; CHD, Coronary heart disease*.

### Morphologic Factors

Size and DPcomA in Unruptured PcomA-BAs were significantly larger than those in PcomA-IDs (Table 2); however, the APcomA and AICA in the PcomA-BAs group were significantly lower than those in the unruptured PcomA-IDs group. There were no significant differences with respect to the other six morphological parameters (Table 2).

**Table 2:** Results of hemodynamic and morphological parameters.

**Varaiable**	**PcomA-IDs**	**PcomA-BAs**	***P*-values**
Size	2.36 ± 0.55	4.42 ± 1.57	<0.001
AR	1.02 ± 0.19	1.07 ± 0.24	0.10
SR	1.48 ± 0.33	1.58 ± 0.57	0.12
EI	0.11 ± 0.03	0.12 ± 0.04	0.06
UI	0.046 ± 0.01	0.049 ± 0.02	0.16
NSI	0.13 ± 0.03	0.14 ± 0.05	0.13
DICA	3.31 ± 0.38	3.23 ± 0.43	0.17
DPcomA	1.04 ± 0.25	1.37 ± 0.33	<0.001
APcomA	79.53 ± 12.77	42.95 ± 15.82	<0.001
AICA	137.86 ± 12.08	132.02 ± 14.91	0.004
NWSS	0.81 ± 0.09	0.76 ± 0.14	0.01
OSI	0.0092 ± 0.0043	0.010 ± 0.0055	0.23
RRT	0.14 ± 0.03	0.15 ± 0.05	0.31

*AR, aspect ratio; SR, size ratio; EI, ellipticity index; UI, undulation index; NSI, non-sphericity index; DICA, the diameter of the internal carotid artery; DPcomA, the diameter of the posterior communicating artery; APcomA, the angle between the internal carotid artery and posterior communicating artery; AICA, the angle between the ophthalmic and communicating segments of the internal carotid artery; Normalized WSS is the WSS-aneurysm wall divided by the WSS-parent artery; OSI, oscillatory shear index; RRT, relative residence time; PcomA, posterior communicating artery*.

### Hemodynamic Factors

**Figure 1:**
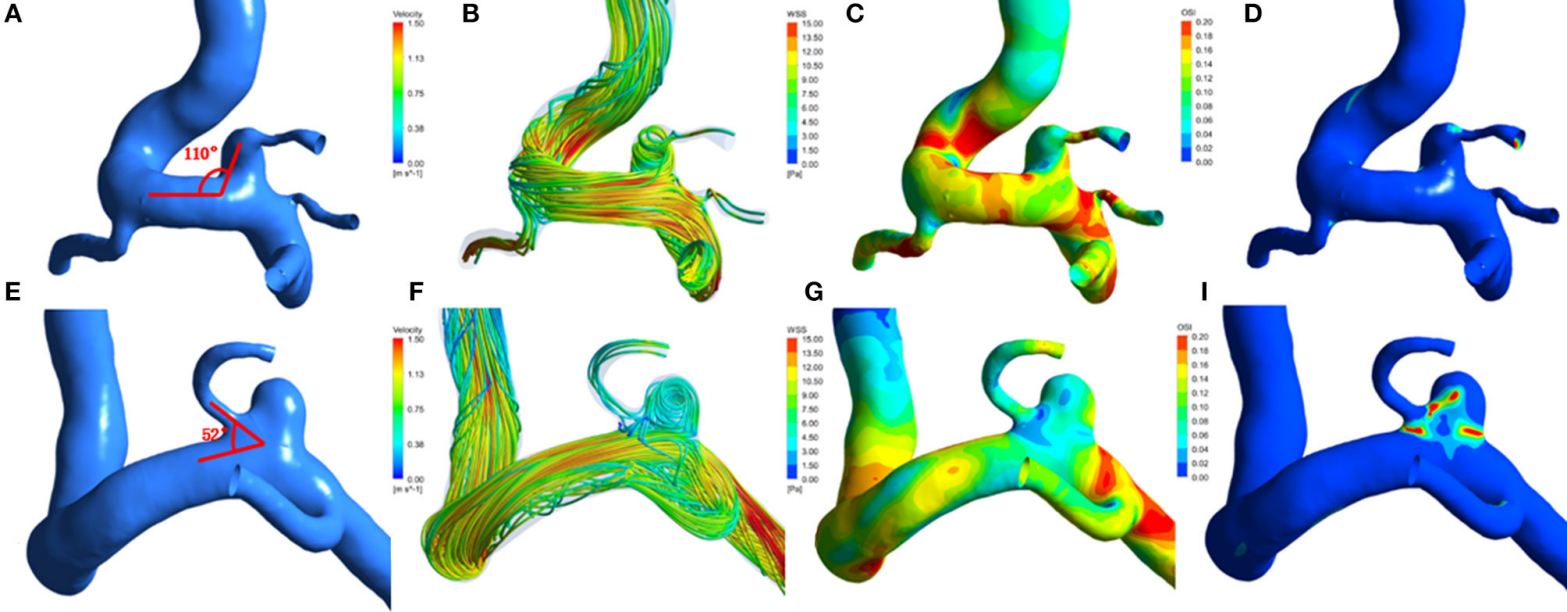
Hemodynamic patterns of a representative infundibular dilation of posterior communicating artery (PcomA-ID) and a unruptured carotid-posterior communicating artery bifurcation aneurysm(PcomA-BA). **(A)** PcomA-ID with a higher angle between the ophthalmic segment of the ICA and the PcomA (ApcomA, 110°); **(E)** unruptured PcomA-BA with a lower ApcomA (52°). From left to right: **(A,E)** Three-dimensional models and measurement of ApcomA; **(B,F)** flow pattern at the systolic peak. **(C,G)** distribution of wall shear stress(WSS); **(D,I)** distribution of oscillatory shear index(OSI).

A total of 198 patient-specific models were reconstructed. The distributions of was in the PcomA-IDs and the unruptured PcomA-BAs are shown in Figure 1.

Compared with the PcomA-IDs, the unruptured PcomA-BAs group had significantly lower NWSS (0.76 ± 0.14 vs. 0.81 ± 0.09 *p* = 0.001). Despite the fact that the unruptured PcomA-BAs usually had higher OSI (0.010 ± 0.0055 vs. 0.0092 ± 0.0043) and RRT (0.15 ± 0.05 vs. 0.14 ± 0.03) than those in the PcomA-IDs, there were no significant differences between the groups (Table 2, Figure 1).

### Binary Logistic Regression and ROC Analysis

Three morphological parameters (size, ApcomA and DpcomA) were independent predictive factors for the status of PcomA-IDs (Table 3).

**Table 3:** The results of binary logistic regression analysis.

**Variable**	**OR (95% CI)**	***P*-values**
APcomA	0.856 (0.805, 0.910)	<0.001
SIZE	5.402 (2.161, 13.504)	<0.001
DPcomA	19.195 (1.630, 225.997)	0.019
AICA	0.990 (0.945, 1.037)	0.673
NWSS	0.002 (0.000, 3.271)	0.101

The ORs indicated that unruptured PcomA-BAs were 5.402 times (95% CI 2.161 to 13.504, *p* < 0.001) more likely to be larger in size and 19.195 times (95% CI 1.630 to 225.997) more likely to have greater Dpcom. ApcomA (OR = 0.856, 95% CI 0.805 to 0.910, *p* < 0.001) was inversely correlated with unruptured PcomA-BAs.

**Figure 2:**
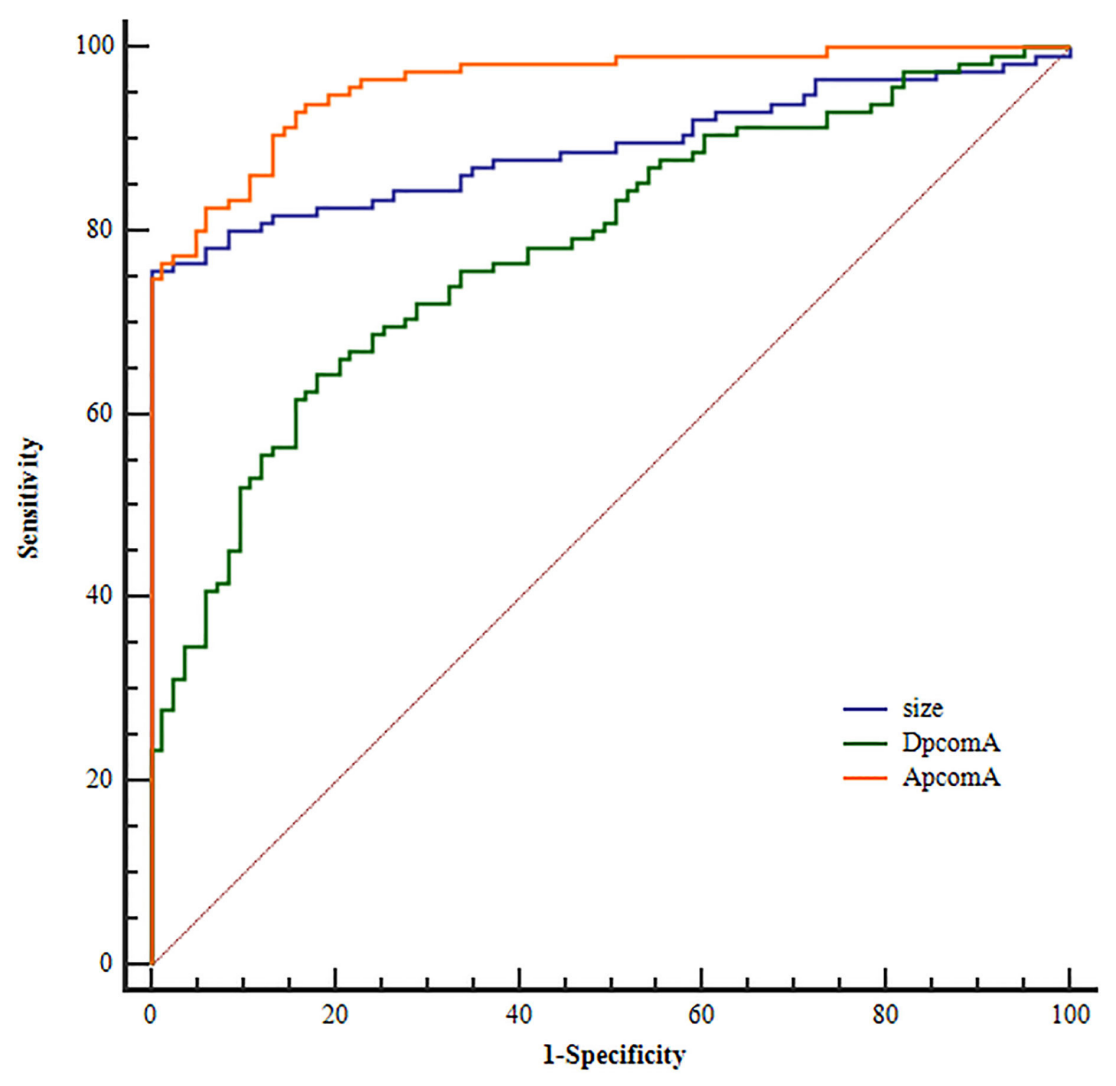
Receiver operating characteristic (ROC) curves for independent risk factors.

ROC analysis for independent risk factors indicated that the cutoff values of size, APcomA, and DPcomA were 3.45 mm, 66.27°and 1.24 mm, respectively (Figure 2).

## Discussion

We considered three hemodynamic parameters and ten morphological parameters, evaluated using patient-specific PcomA-ID and PcomA-BA models, to distinguish the PcomA-IDs from PcomA-BAs. We found that size, DpcomA, and ApcomA were independent parameters to characterize the status of PcomA-IDs.

Although a substantial number of morphological factors have been introduced to distinguish the status of IAs (24–26), size is the most important morphological factor used in routine clinical practice to guide treatment or management. Studies of unruptured intracranial aneurysms showed that size is an important predictor of rupture; aneurysms larger than 7 mm are at higher risk for SAH (27, 28). Akio et al. found that, with an annual rate of rupture of 0.95%, the risk of rupture increased with increasing size of the aneurysms (27). Jiang et al. compared morphological parameters using 14 mirror posterior communicating artery aneurysms and found that larger size may be related to rupture of PComA aneurysms (29). In our study, size was also a parameter that distinguished PcomA-IDs from PcomA-BAs.

The DpcomA, defined by averaging the diameter of the cross-section of a vessel (D) just proximal to the neck of the aneurysms or IDs and the diameter of the cross-section at 1.5 times D from the neck of the aneurysms or IDs in our study, may differentiate between PcomA-IDs and PcomA-BAs. Ebina et al. found the PcomA-IDs with well-developed PcomAs (large DpcomAs) were more likely to become aneurysms (2). Endo et al. treated 32 patients with 34 lesions of enlarged PcomA-IDs (30); they found that six of the lesions became true PcomA-BAs and large PcomAs were established in all six lesions. These results are consistent with those of our study.

The ApcomA in our study is the angle between ICA and PcomA. In previous studies, the inflow angle, defined as the angle between inflow and the main axis of aneurysm from the center of the neck to the tip of the dome, was associated with rupture of aneurysms (21, 31, 32). Baharoglu et al. found that, with increasing IA, the flow vortex zone migrated closely to the aneurysm tips and higher peak velocities and kinetic energy transferred into the distal part of the aneurysms dome as well as the WSS and WSS gradients of dome and inflow zone became higher (31). Agreeing with the results that Baharoglu et al. acquired, Lv et al. studied morphological parameters in small posterior communicating artery aneurysms and found that SR and IA were independent parameters for stratification of small PcomA aneurysms (33); ApcomA was similar to IA. For PcomA-IDs, the ApcomA was equal to IA. Yu et al. argued that the APcomA could distinguish normal arteries from internal carotid–posterior communicating artery aneurysms (22). The values in the internal carotid–posterior communicating artery aneurysms (79.22 ± 17.83) were significantly higher than the values in normal arteries (45.28 ± 29.07). The present study also showed that the APcomA in PcomA-IDs was significantly higher compared to those in the PcomA-BAs.

Although the study by Yu et al. reported that APcomA might correlate with the formation of an internal carotid artery–posterior communicating artery aneurysm, several potential problems ignored by them might cast doubt on their results: firstly, the internal carotid artery–posterior communicating artery aneurysms were not divided into ruptured and unruptured ones; secondly, they only included nine normal arteries and nine internal carotid artery–posterior communicating artery aneurysms and the sample size was small. Finally, hemodynamic factors play important roles in the pathogenesis, progression, and rupture of IAs. In their study, morphological parameters were analyzed; however hemodynamic parameters were not included. By contrast, the present study might provide reasonable results.

As the ApcomA decreased, inflow velocity became relatively higher and flow velocity acceleration produced a relatively high WSS and a positive WSS gradient along the flow, possibly initiating cascades of biochemical signals in the vessel wall through endothelium-mediated mechanotransduction, leading to endothelial dysfunction and disruption of the internal elastic lamina, and finally triggering aneurysm initiation (15, 34, 35). Although the high WSS and positive WSS gradient might be responsible for the aneurysms formation, most aneurysms developed to low WSS state after aneurysm initiation. Cebral et al. analyzed the local hemodynamics and the formation of blebs in cerebral aneurysms based on CFD (10). Their findings are consistent with those of our study to the effect that the WSS in PcomA-Bas (0.76 ± 0.14 pa) was significantly lower than the WSS in PcomA-IDs (0.81 ± 0.09 pa). Nevertheless, the difference did not reach statistical significance in binary logistic regression analysis.

There are some potential limitations in our study. Firstly, to compare the hemodynamic and morphological parameters between the PcomA-IDs and PcomA-BAs, the PcomA-IDs were treated as PcomA-BAs. Secondly, for the development of PcomA-IDs, according to current literature review, they all developed into PcomA-BAs, but they may also developed into sidewall PcomA aneurysms. Thirdly, to establish the patient-specific PcomA-IDs and PcomA-BAs models, some small vessel branches far away the aneurysms were artificially moved. Fourthly, while the study was a retrospective analysis and patient-specific models were applied in the CFD simulation, the inlet boundary conditions were not patient-specific and the assumptions of laminar flow, Newtonian blood and rigid wall were used in our hemodynamic simulation. Doing so may lead to inaccurate results and less accurate conclusions. Fifthly, all the patients in this study were treated at a single center and the sample size was relatively small. Further multi-center studies with larger sample size are required to validate our results. Finally, Because of the complexity and time-consuming process of current CFD research as well as multiple variables being left out of the equation, the interpretation of this study is complicated and its application in clinical work is limited. CFD simulation needs to be simplified and some equations need to be developed for use in measurement of multiple variables in future studies to allow decision-making in a timely fashion and make the studies easier to interpret.

## Conclusion

We compared three hemodynamical (e.g., TWSS, RRT) and ten morphological (e.g., size, Apcom) parameters between PcomA-IDs and PcomA-BAs. Size, DpcomA, ApcomA were found to be independent parameters to characterize the status of PcomA-IDs. These might help us to better manage PcomA-IDs. We hope that these results will provide data to guide further studies and appropriate treatment strategies.

## Data Availability Statement

All datasets generated for this study are included in the article/supplementary material.

## Ethics Statement

The Research Ethics Committee of Wannan Medical College approved this retrospective study; informed consent was acquired. All patients were treated at the Department of Neurosurgery, the First Affiliated Hospital of Wannan Medical College, Wuhu City, China.

## Author Contributions

JY, XF, and CH contributed the conception, design, and drafted the manuscript. XJ, ZL, and XZ collected the data and data analysist. DW, NL, and JL made critical revisions to the manuscript. XW and DX contributed to final approval of the version to be published. All authors contributed to the article and approved the submitted version.

## Conflict of Interest

The authors declare that the research was conducted in the absence of any commercial or financial relationships that could be construed as a potential conflict of interest.
